# MIPHENO: data normalization for high throughput metabolite analysis

**DOI:** 10.1186/1471-2105-13-10

**Published:** 2012-01-13

**Authors:** Shannon M Bell, Lyle D Burgoon, Robert L Last

**Affiliations:** 1 Quantitative Biology Program, Michigan State University, East Lansing, MI, USA; 2 Department of Biochemistry and Molecular Biology, Michigan State University, East Lansing, MI, USA; 3 National Health and Environmental Effects Research Laboratory, Biostatistics and Bioinformatics Research Core, United States Environmental Protection Agency, Research Triangle Park, NC, USA; 4 Department of Plant Biology, Michigan State University, East Lansing, MI, USA

## Abstract

**Background:**

High throughput methodologies such as microarrays, mass spectrometry and plate-based small molecule screens are increasingly used to facilitate discoveries from gene function to drug candidate identification. These large-scale experiments are typically carried out over the course of months and years, often without the controls needed to compare directly across the dataset. Few methods are available to facilitate comparisons of high throughput metabolic data generated in batches where explicit in-group controls for normalization are lacking.

**Results:**

Here we describe MIPHENO (Mutant Identification by Probabilistic High throughput-Enabled Normalization), an approach for post-hoc normalization of quantitative first-pass screening data in the absence of explicit in-group controls. This approach includes a quality control step and facilitates cross-experiment comparisons that decrease the false non-discovery rates, while maintaining the high accuracy needed to limit false positives in first-pass screening. Results from simulation show an improvement in both accuracy and false non-discovery rate over a range of population parameters (p < 2.2 × 10^-16^) and a modest but significant (p < 2.2 × 10^-16^) improvement in area under the receiver operator characteristic curve of 0.955 for MIPHENO vs 0.923 for a group-based statistic (*z*-score). Analysis of the high throughput phenotypic data from the Arabidopsis Chloroplast 2010 Project (http://www.plastid.msu.edu/) showed ~ 4-fold increase in the ability to detect previously described or expected phenotypes over the group based statistic.

**Conclusions:**

Results demonstrate MIPHENO offers substantial benefit in improving the ability to detect putative mutant phenotypes from post-hoc analysis of large data sets. Additionally, it facilitates data interpretation and permits cross-dataset comparison where group-based controls are missing. MIPHENO is applicable to a wide range of high throughput screenings and the code is freely available as Additional file 1 as well as through an R package in CRAN.

## Background

High-throughput screening studies in biology and other fields are increasingly popular due to ease of sample tracking and decreasing technology costs. These experimental setups enable researchers to obtain numerous measurements across multiple individuals in parallel (e.g. gene expression and diverse plate-based assays) or in series (e.g. metabolomics and proteomics platforms). The large number of measurements collected often comes at the cost of measurement precision or the overall power of detection. For many large-scale studies, the experimental design aims to maximize the number of compounds or individuals tested, resulting in limited replication and few to no controls. In the case of microarray studies, several methods for normalizing arrays have been developed [1-3] with no universal method adopted as the standard. Quantitative PCR faces the same issues as it is used more frequently in high throughput platforms, with analysis methodologies being developed paralleling those for expression arrays [4].

Metabolite profiling is a rapidly expanding area of high throughput measurements, where samples having large amounts of biological variability and diverse physical properties makes quantification of large numbers of structurally diverse metabolites challenging [5]. Few strategies exist for normalization in metabolite analysis to control for run-to-run variance other than to include negative and positive controls. For large-scale screens involving mutagenized populations (plant, bacteria) or crosses (plant breeding), the goal is to identify putative hits, or individuals that are likely to be different from the bulk of the samples for subsequent follow-up (e.g. [6]). In these conditions, properties of the sample cohort serve as controls with the measure of differences between an individual and its cohort used to identify samples differentially accumulating a metabolite [6]. This strategy can streamline sample processing and maximize throughput when the expected effects are large and easily observable.

For studies where comparisons are sought across an experiment conducted over the course of several months or in different sample batches, normalizing factors are necessary, especially given typically high levels of biological and technical variability [7-9]. Ideal experiments include technical and biological replication within each set as well as controls facilitating comparisons between sample batches, but these are often limited or omitted entirely due to likely increases in experimental costs or the negative impacts on throughput. However, absence of these experimental controls limits the ability to handle variability between sample groups (e.g. remove batch effects) making it a greater challenge to identify individuals within the range between normal and aberrant phenotypes. Without the ability to normalize the data provided by experimental controls, some of the benefits of high throughput screens are lost, yet the desire to maximize throughput places constraints on the experimental design.

The motivation for algorithm development came from the *Arabidopsis thaliana *Chloroplast 2010 Project large-scale reverse genetic phenotypic screen (Chloroplast 2010, http://www.plastid.msu.edu/, [10-13]). This project leverages the collection of T-DNA insertion lines and genomic sequence for the plant model species *A. thaliana *to screen large numbers of putative gene knockouts with the aim of functionally characterizing chloroplast-targeted genes. The presence of a large T-DNA insertion can block or reduce expression of the gene it lands in, and altered phenotypes can provide insights into the normal function of the gene and its protein or RNA product(s).

In addition to qualitative and semi-quantitative measures of physiological and morphological characteristics, the pipeline assayed levels of leaf fatty acids and leaf and seed free amino acids, important outputs of chloroplast metabolism. The pipeline assays were preformed on groups of individual plants planted in units of up to thirty-two per tray and three trays of plants per assay group. Two assay groups were grown concurrently under controlled environment plant growth conditions. Individuals representing T-DNA insertion events in different locations within the same gene (alleles) are present in the dataset, and it is of interest to compare the assay responses of these individuals as well as to identify other individuals with similar responses. Because the experimental design lacked cross-group controls (e.g. designated WT), the ability to make even semi-quantitative cross-dataset comparisons was not possible using existing methodology.

Developing phenotypic annotation for un- and under- annotated genes is a primary goal for the Chloroplast 2010 project and identification of individuals with like phenotypes (phenotypic clustering) is a way to achieve that goal. Thus, a method that would allow cross-dataset comparisons and identify putative mutants was needed to achieve the goal. The resulting method, MIPHENO (Mutant Identification by Probabilistic High throughput-Enabled Normalization), is aimed at improving first-pass screening capabilities for large datasets in the absence of defined controls. Algorithm performance was tested using a synthetic data set and the Chloroplast 2010 high throughput phenotypic dataset. The executable code and data for the Chloroplast 2010 analysis are available as Additional file 1 and as a CRAN package (MIPHENO, http://cran.r-project.org/web/packages/MIPHENO/index.html).

The following describes a quality control process for identifying aberrant groups followed by a data normalization method, which aims to bring samples into the same distribution allowing for dataset-wide comparisons. Additionally, we describe a hit detection function based on the cumulative distribution function (CDF) to identify samples with putative, 'non-normal' phenotypes. For clarity, the terms normal and wild type (WT) are used to describe the typical response of the population. Generally, this could be the untreated (chemically or genetically) population or the base level of the system (e.g. background response). Non-wild type responses, a hit or mutant, refer to a response that is distinct from the normal response distribution, with a putative hit/putative mutant referring to a sample that is predicted to have a response different from the normal response distribution but has yet been confirmed. In high throughput screens, the objective is to identify putative hits balancing the false positive rate (FPR), or the number of WT samples that are called hits, with the false non-discovery rate (FNDR), the number of true hits that are missed. Results are presented from analysis of the synthetic dataset and biological data.

## Results

### Input Data Characteristics and Structure

MIPHENO is specifically designed for the analysis of first pass screening data where the majority of measured responses are from the WT or normal class and the number of responses not in this group (putative hits) is quite small. Examples of experiments yielding appropriate data are non-targeted protein binding/activator assays, reporter gene assays, or population screens, where there are either no defined classes or very unbalanced classes such that a large majority of responses fall in the WT class. Data coming from a treatment vs control experiment would not meet the criteria if there were large numbers of 'non-WT' responses expected. Additionally, the approach is tolerant to repetition of both individual samples and sample groups across the course of the experiment so long as the portion of individuals showing a WT response in any sample group is over 50%. As the portion of WT individuals in a sample group decreases, there will be a reduction in accuracy and a corresponding increase in false non-discovery rate (FNDR) due to the assumptions of the algorithm, as demonstrated in the Testing section below. Additionally, while some measured responses may not be independent (ex, metabolite measures of branch chain amino acids), the method treats these attributes (e.g., metabolites) as independent to increase the flexibility of the analysis. For instance, the results for attribute 1 (including normalization and downstream analyses) do not impact the results for attribute 2. This is beneficial in post hoc analysis where the individual performing the analysis has limited knowledge of the relationship between measures.

Input data for analysis by MIPHENO assumes that multiple attributes are measured for each individual. The data structure treats each row as an individual sample, whose relationship to other samples can be described by one or multiple factor variables represented in columns (grouping factor). For example, the assay group representing the identification number for a 96-well plate containing up to 96 individuals. Subsequent columns describing the response of the individual to some assay (attribute response) are quantitative, continuous values. Information must be present that enables association of a grouping factor to the attribute responses, but a single data object may include the responses for different attributes as long as the appropriate grouping factor is present. For example, a 'LC_ID' column might provide the grouping factor for ten columns of LC-MS amino acid data, while 'HPLC_ID' might provide the grouping factor for five columns of HPLC-derived responses on the same set of samples. This structure is aimed at simplifying situations where multiple measurements are taken on the same individual.

### Algorithm

MIPHENO is based on invariant set normalization with three key assumptions made of the input data. The first is that samples from the same genetic background should have a similar assay response over time. This implies that, given a population P, the distribution of an observed response r from sample set p in set P should have the same distribution as the response R from population P as p approaches P. Following this logic, the second assumption of the data is that the observed differences between the distributions r and R are due to technical error as opposed to biological or genetic variance as p approaches P. The last assumption is that there will be limited observable effects of simple genetic manipulations to an organism for any random gene. This is based on empirical evidence from years of published studies [6,14-16]. Specifically, due to genetic redundancy and metabolic flexibility, a given disruption in gene function will likely cause a response outside the WT distribution in only a limited number of measured responses.

These assumptions are similar to those for microarray analysis, specifically that for a random or large grouping of individuals (e.g. cDNAs), changes will be observed for a relatively small proportion [17]. Other assumptions used to normalize the data (e.g. a balance in the total amount of transcript in quantile normalization [1]) have the same effect of forcing the median value of a sample set across several experiments or arrays to be equal. Similar assumptions also apply to data from other high throughput screens, e.g. reporter gene-based assays and enzymatic assays.

**Figure 1:**
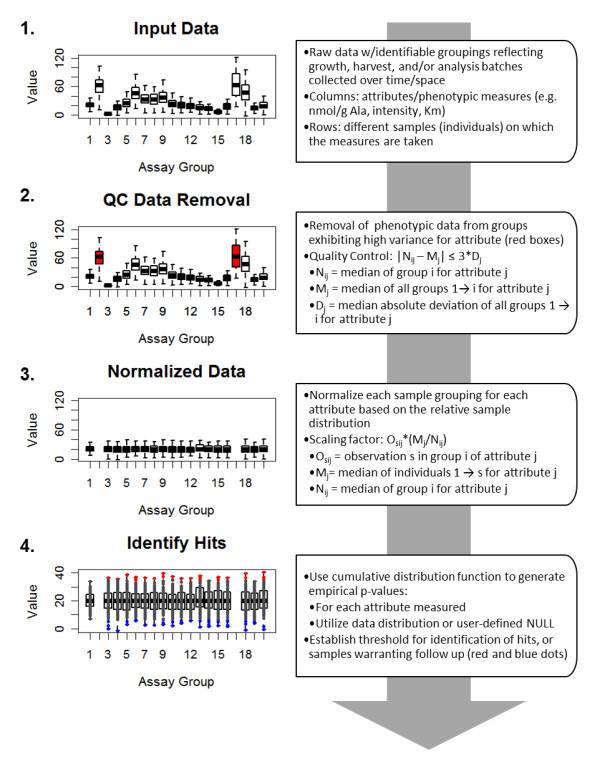
**Flowchart of MIPHENO**. "Input Data" (1) contains data with identifiable parameters for grouping/processing the data. The data pass through a quality control (QC) removal step (2), where groups not meeting the cut offs are identified and removed on an attribute-by-attribute basis. Data are normalized (3) using a scaling factor based on the data distribution. Putative hits are identified (4) using a CDF built from the data or user defined NULL distribution and an empirical *p*-value is assigned to each observation. Thresholds can be established based on follow-up capacity and prior knowledge (e.g. ability to detect known 'gold standard' mutant samples).

An overview of the algorithm is presented in Figure 1. The algorithm requires that input data have a grouping factor that presents a batch or process group on which the normalization steps can be performed (see "Input Data Structure and Characteristics" above). If multiple grouping factors are present (e.g. different sample collection, processing, and analysis dates) it is recommended to use the factor representing the highest level of technical (i.e. non-biological) error for normalization. This can be determined by familiarity with the methodology or by checking the grouping factors to see which factor has the largest interquartile range for group medians.

### Quality Control Method

In performing post-hoc data analysis it is often unknown if on-line quality control (QC) was conducted or where process changes occurred that could negatively affect the outcome of analysis. To address these issues, a quality control (QC) step prior to analysis was included to identify samples with a high likelihood of assay or group-specific process error. Examples of sources of these types of error include instrument malfunction (for assay-specific error), abnormalities in growth or preparation of material (group-specific error), or improper sample handling affecting a group of samples exposed to the same conditions rather than an individual response. If an on-line QC step was already used to filter the dataset this step can be omitted. Thresholds for QC are determined from the overall distribution of the collected data with a user-defined cut off; for example groups with group median > 3 median adjusted deviations (MAD) from the global median. The amount of data removed will depend on the cut off used and the data distribution. A visual inspection of the data using box and whisker plots is advised to check the data for clear signs of drift or likely changes in protocol that may require manual QC. Examples would be group medians steadily increasing or decreasing across dataset or a switch to a new average median response corresponding with sample order, respectively. For post hoc analysis on datasets where the order in which samples were assayed or collected is unknown, it may be advisable to use a cut off of 3 MAD to permit more data passing on to the next stage.

Data quality is assessed on an attribute-by-attribute basis with the assumption that the measured traits are independent; with an attribute being any measured or observed response. Thus, if multiple attributes are measured for a group (for example, numerous metabolites or promoter-reporter gene outputs), only attribute data for the trait that shows high deviation would be removed and the rest of the data for the group retained. For example, 'HPLC_ID' is the grouping factor for the response of metabolites, such as amino acids. The overall response distribution of each metabolite is assumed to be independent of the other metabolites; thus if the measured response of alanine is 10x the response of proline it will not impact the QC step (or subsequent steps). If the median response for alanine in HPLC_ID = 1 is greater than the QC cut off, all responses for alanine in HPLC_ID = 1 are removed but the other measured responses for HPLC_ID = 1 are retained, provided they too pass QC. While this does not control for drift, it provides a facile QC step for post-hoc data analysis where the order of data generation is unknown.

### Normalization

The normalization process is done on an attribute-by-attribute basis using a user-defined grouping. A grouping factor should encompass the highest amount of non-biological variation and may be the same factor used in the QC step, but should include as many individuals as possible (e.g. n > 10). A scaling factor is calculated to bring the median of each group to the global median, similar to invariant set normalization [4]. The key difference from invariant set or quantile strategies is that just the median value is used, not an explicit individual or multiple quantiles to take into account lack of replication between groups and limited sample size. It is important that groupings represent a selection of individuals where the frequency of non-WT behaviours approaches that of the overall population to avoid bias in cases when a particular group is enriched with non-WT behaviors for a given attribute.

### Testing

**Figure 2:**
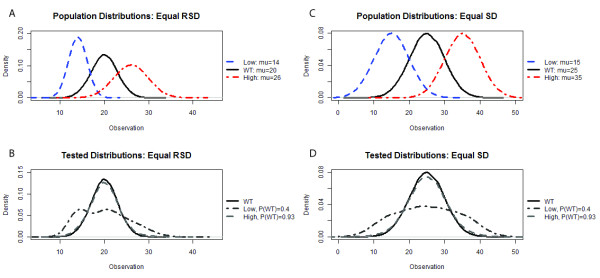
**Synthetic Populations used in Testing**. Synthetic data were generated to measure the performance of the three different methods in a case where 'ground truth' is known. Samples were randomly drawn from a low abundance population (Low, blue line), high abundance population (High, red line) or a WT population (WT, black line) as shown in the upper panels (A, C). Two population structures were sampled, one with a low probability of WT, P(WT = 0.4), and the other with a high probability of WT, P(WT) = 0.93, shown in the lower panels (B, C). To test the effect of population shape, equal relative standard deviation (RSD = 15%, A and B) or equal standard deviation (SD = 5, C and D) were independently tested.

To gauge the performance of the approach, a synthetic dataset was generated emulating characteristics of actual data (see Methods). This dataset was used initially since the true properties of the individuals could be known, allowing for observation classification (e.g. WT and mutant) and to evaluate the effect of population distribution on the performance of the method. Figure 2 illustrates the population distributions used to test the performance of MIPHENO.

**Figure 3:**
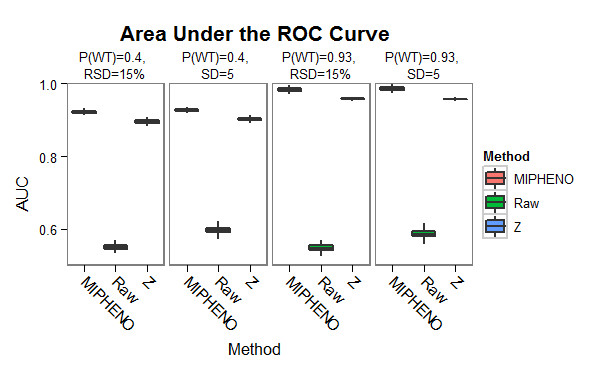
**Performance of Methods on Synthetic Data: AUC**. The AUC was used to evaluate classification performance of MIPHENO, the use of raw data followed by a CDF classifier (RAW), and a group-based metric (Z) on synthetic data described in Figure 2. MIPHENO (pink, first in set) outperforms both RAW (green, middle) and Z (blue, left in set) across the different population parameters.

**Figure 4:**
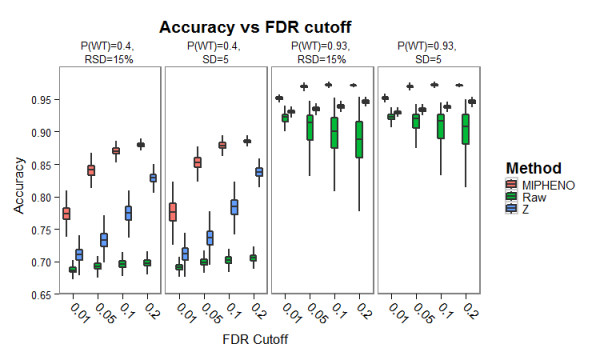
**Performance of Methods on Synthetic Data: Accuracy**. Accuracy of classification was used to compare the performance of MIPHENO, the use of raw data followed by a CDF classifier (RAW), and a group-based metric (Z) on synthetic data from populations described in Figure 2. The percent accuracy is plotted along the y-axis while the false discovery rate (FDR) cut off is along the x-axis. Each population distribution tested is shown in a separate panel. Note that MIPHENO (pink) achieved higher classification than Z (blue) (p < 2.2e-15, Wilcoxon sign rank) and both methods outperformed Raw (green) independent of the population parameters tested.

**Figure 5:**
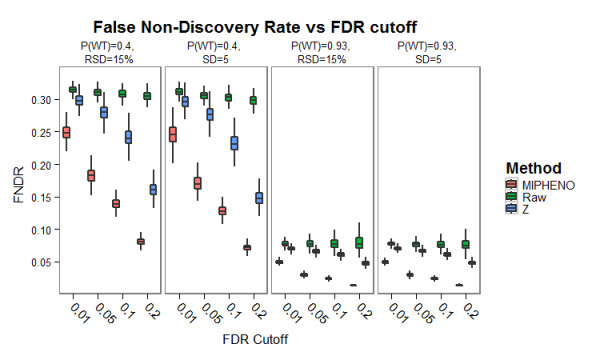
**Performance of Methods on Synthetic Data: False Non-Discovery Rate**. The false non-discovery rate (or percent positive hits missed) was used to compare the performance of MIPHENO, the use of raw data followed by a CDF classifier (RAW), and a group-based metric (Z) on synthetic data from populations described in Figure 2. The FNDR is plotted along the y-axis with the different false discovery rate (FDR) cut offs along the x-axis. Each population distribution is shown in a different panel. Note that across all populations tested, MIPHENO has a lower FNDR than the other two method, suggesting that fewer putative hits will missed with MIPHENO compared to using the Z-score (blue) or raw data (green).

Comparison of two different data analysis approaches was used to test 1) if pre-processing steps remove high amounts of real biological variation indicative of a putative hit and 2) whether an increased false non-discovery rate (FNDR) resulted from using MIPHENO verses a sample-group based method (results in Figures 3, 4, and 5). The first approach referred to as 'Raw', uses the raw, unprocessed data, but followed the same process as in MIPHENO to identify putative mutants. Differences between Raw and MIPHENO aid in illuminating the effectiveness of pre-processing in noise removal. The second approach, referred to as 'Z', also utilized the raw data but used a MAD score on a sample-group basis to identify putative mutants as described for the Chloroplast 2010 data [10]. Comparison of MIPHENO to Z aids in determining potential loss of information due to normalizing across the data sets (e.g. whether true mutants were more severely scaled in normalization), or if the group-based error was controlled for without negatively impacting hit detection. In a review of performance metrics by Ferri et al. [18], accuracy (ACC) was found to be a better metric than area under the receiver-operating curve (AUC) in the case of unbalanced sample size as well as misclassification noise, which are both properties of the data under analysis. Conversely, they found AUC outperformed ACC in probability and, to a lesser degree, ranking noise. False non-discovery rate is an important metric when considering first-pass screens as one seeks to limit the true positives missed, which is the situation described here.

Results of the performance trials using a combination of two population distributions that had a high frequency of WT (P(wt) = 0.93) and low WT frequency (P(wt) = 0.40), drawn from populations of equal standard deviation (SD) or relative standard deviation (RSD) (Figure 2), are shown in Figures 3, 4, and 5. These results suggest that the proportion of true WT in the sample had little effect on the performance of the methods relative to each other, regardless of the metric used; however, the accuracy is decreased and the false non-discovery rate is increased for all methods when the portion of data from the mutant class is increased (Figures 4 and 5). MIPHENO showed a higher accuracy and lower FNDR (p < 2.2 × 10^-16^, Wilcoxon signed rank test) across a range of FDR cut offs compared to the other methods (Figure 5). Furthermore, the AUC of both MIPHENO and Z outperformed an analysis of Raw (Figure 3), which performed just above what is expected at random, highlighting the importance of controlling for group-based variability. In summary, MIPHENO outperformed both the Raw and Z-methods across all three metrics tested.

### Implementation

**Figure 6:**
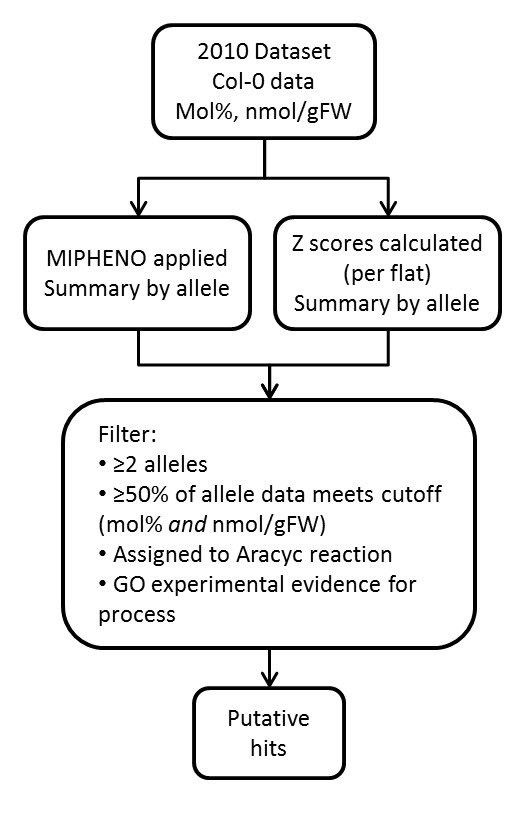
**Flowchart of Performance Measures for Chloroplast 2010 Data**. Metabolite data from wild-type Col-0 ecotype samples were taken from the Chloroplast 2010 dataset. MIPHENO empirical *p*-values and *z*-scores were calculated separately for metabolite values reported as mol % and nmol/g fresh weight (nmol/gFW) and results filtered according to criteria. Publicly available annotation (Aracyc and GO, Additional file 1) for annotated genes provided a basis of comparison between the two metrics.

Results from the Chloroplast 2010 Project [10,11] were used to test the performance of MIPHENO on experimentally generated high throughput screening data. This dataset includes results for leaf protein amino acids and fatty acid methyl esters as well as seed protein amino acids for plants run through the Chloroplast 2010 pipeline. Multiple individuals representing the same seed stock or the same gene are present in the dataset although they were not assayed in the same group. Thus, it is of interest to look at the consistency between individuals representing the same gene to identify Leaf and seed metabolite data from mutants in the Col-0 (CS60000, [19]) ecotype genetic background were processed using MIPHENO and *z *score methods independently. Figure 6 outlines the methods for comparison. Briefly, both MIPHENO empirical *p*-values and *z *scores were calculated for the two data measurements available in the Chloroplast 2010 dataset (mol% and nmol/gFW). The average score per T-DNA insertion line was calculated for each data type to avoid overemphasizing lines that were analyzed multiple times. Aracyc [20] and Gene Ontology (GO) [21] information obtained from The Arabidopsis Information Resource (TAIR) [22] were used to generate a list of loci previously demonstrated to have a biological function in Arabidopsis. Loci with phenotypes predicted by the methods were compared to the list of literature-documented loci. The biological role and/or phenotypes of the genes were compared to the published information to determine the accuracy of the prediction. Results are given in Table 1. While both methods had a similar frequency of correctly identifying mutant phenotypes at the initial level of Z cut off of 2.5, the Z method returned fewer lines than MIPHENO. It was necessary to adjust the Z threshold to 1.3 to recover these lines, which resulted in no additional mutants but an increase in false positives. Overall, there was ~four-fold improvement in the ability to detect previously described or expected phenotypes compared with the *z-*score.

**Table 1:** Lines identified by MIPHENO and Z methods

Locus	Description	Tissue	MIPHENO Cutoff = 0.1	Zscore Cutoff = 2.5	Zscore Cutoff = 1.3
**At1g08250**	ADT6: Plastid-localized	Seed	**High**: GLN, TYR		**High**: GLN, TYR
		
	arogenate dehydratase	Leaf			

**At1g09795**	ATATP-PRT2: ATP	Seed			
		
	phosphoribosyl transferase	Leaf	**High**: HIS		**High**: HIS, LEU

**At1g11790**	ADT1: Plastid-localized	Seed			
		
	arogenate dehydratase	Leaf	**Low**: PHE		**Low**: PHE

**At1g65960**	GAD2: glutamate	Seed			**Low**: GABA
		
	decarboxylase	Leaf	**Low**: GABA	**Low**: GABA	**Low**: GABA

**At2g39800**	P5CS1: delta1-pyrroline-	Seed	**Low**: HPRO		
		
	5-carboxylate synthase	Leaf	**Low**: PRO		**Low**: PRO

**At3g11170**	FAD7: Responsible for the synthesis of 16:3 and	Seed			
		
	18:3 fatty acids	Leaf	**High**: 16:1D7, 16:2, 18:1D9, 18:2; **Low**: 16:3, 18:3	**High**: 16:2, 18:1D9, 18:2; **Low**: 16:3, 18:3	**High**: 16:1D7, 16:2, 18:1D9, 18:1D11, 18:2; **Low**: 16:3, 18:3

**At3g45300**	IVD: Isovaleryl-CoA	Seed	**High**: ARG, GABA, HIS, ILE, LEU, MET, TRP, VAL; **Low**: GLU	**High**: ARG, GABA, HIS, ILE, LEU, TRP, VAL, MET; **Low**: GLU	**High**: N, ARG, GABA, HIS, ILE, LEU, LYS, MET, PRO, SER, TRP, TYR, VAL; **Low**: GLU
		
	Dehydrogenase	Leaf	**High**: 16:3; **Low**: 18:2		**High**: 16:3, GABA; **Low **18:2

**At4g19710**	AK-HSDK II: Bifunctional aspartate	Seed			
		
	kinase, homoserine dehydrogenase.	Leaf	**High**: 18:1D11, CYS, HSER, ILE, THR	**High**: CYS, HSER, ILE, THR	**High**: 18:1D11, CYS, HSER, ILE, THR

At4g27030*	FAD4: Palmitate	Seed			
		
	desaturase	Leaf	**High**: 16:0, ALA, GLN, L.ALA; **Low**: 16:1D3	**High**: ALA; **Low**: 16:1D3	**High**: 16:0, ALA, GLN, SER, TRP; **Low**: 16:1D3

**At4g33150**	LKR/SDH: Splice variant of a bifunctional	Seed	**High**: HIS, LYS; **Low**: GLU	**High**: HIS, LYS	**High**: HIS, LYS, PRO
		
	enzyme for lysine catabolism	Leaf			

**At5g05730**	ASA1: Alpha subunit of	Seed			
		
	anthranilate synthase	Leaf	**Low**: TRP	**Low**: TRP	**Low**: TRP

**At5g53460**	GLT1: NADH-dependent glutamate	Seed	**High**: ASN; **Low**: ASP		**High**: ASN, CYS; Low: ASP
		
	synthase	Leaf			

*Aracyc information not updated, manually added

Results of the analysis presented in Figure 6.

## Discussion

MIPHENO offers a way to control for assay variability in high throughout mutant screening studies. It outperformed using raw data or the group-based Z method in mutant identification on the synthetic data set (Figures 3, 4, and 5). Comparison of population parameters including proportion of WT and the distribution shape suggest that the method is tolerant to uneven distributions (tailing) and to higher mutant frequencies within the population. When applied to a biological data set, MIPHENO led to identification of more true mutants than the Z method for the Chloroplast 2010 set (Table 1) based on literature reported phenotypes or pathways. This suggests that MIPHENO reduces the false positive rate by decreasing the variation due to batch effects but does not directly influence the false non-discovery rate. The method additionally offers the user the ability to utilize any *a priori *information on the WT population/NULL distribution available as well as customize a quality control step that is sensitive to the needs of their process.

One drawback of using the normalization strategy described here is that it fails to control for the within-group variance to the degree that a quantile normalization strategy might. Quantile normalization makes the assumption that both the median or mean and the standard deviation of the data are all equal and would require sample sizes to be more or less equal as well as large enough to start approximating the normal distribution. This assumption does not always apply to post-hoc analysis; for example, the size of the sample groups in the Chloroplast 2010 data set varied from 12 to 96. MIPHENO aims at addressing this type of use case.

## Conclusions

The strong performance of MIPHENO on two different data sets and its ability to permit cross-dataset comparisons of individuals without explicit controls makes it an ideal method for processing large datasets prior to Meta analyses combining different data sets from high-throughput experiments. Because more researchers are making their primary data available and the number of large-scale, high-throughput experiments keeps increasing, MIPHENO will provide a valuable processing platform that can theoretically be applied to very diverse measurement types (e.g. gene expression, enzyme kinetics, metabolite amounts).

## Methods

### Data analysis

All calculations were performed in R [23] v 2.11.0 on 64-bit Windows 7 platform with the code available in Additional file 1, 'Code' subfolder with data used in the 'Data' subfolder. Chloroplast 2010 Project data used in the reported analysis was obtained on 8/18/2010. GO and Aracyc pathway information were obtained from the TAIR FTP site, files dated 8/2/2010 and 6/21/2010 respectively (Additional file 1, Data subfolder).

### Generation of synthetic test data

Synthetic data were generated by sampling from three random Gaussian distributions representing low abundance, high abundance, and wild type levels of 'metabolite' (Figure 2) using a set of sampling probabilities (Additional file 1). Distributions were created to assess the effects of uniform variance (e.g. same standard deviation) and proportional variance given by a relative standard deviation of 15% based on prior observations of real data from the Chloroplast 2010 study. Means for the distributions were set such that the means of the 'mutant' populations were two standard deviations away from that of the wild type, because this is a common cut off for identifying hits in screening assays. The proportion of individuals sampled from each population (low, wt, high) was set prior to generating sample groups to test how different population composition influenced algorithm performance. To mirror the biological population structure, data were assigned to a flat, assay, and planting group representing individuals grown in the same physical unit, processed and assayed together, or grown over the same time course, respectively. Classification of each observed value was done at this step, prior to adding random noise (described below), defining a 'low' mutant as one that was 2 standard deviations below the WT mean and a 'high' mutant as one that was 2 standard deviations above. For calculating performance metrics, only the WT and mutant class were considered.

To simulate the non-biological variance, random uniform noise was added first at the level of planting group then at the level of assay group as empirical evidence suggested a greater assay effect than planting group effect. The resulting synthetic dataset was defined as raw data for use in the Z and raw data methods.

### Method Performance using the Chloroplast 2010 data

An overview of the data analysis approach is depicted in Figure 6. Data from the Chloroplast 2010 for mol% and nmol/g FW fatty acid methyl esters and amino acids were used to calculate both MIPHENO empirical *p*-values and *z*-scores. Data used is available as additional files under the 'Data' folder and the script used in the analysis is in the 'Code' folder. Samples genotyped as wild type or heterozygous for the T-DNA insertion were removed. The average phenotypic score (*z*-score or empirical *p*-value) per T-DNA insertion line was calculated and this was used to define the phenotype for that insertion line. Next, loci where there were ≥ insertion lines showing the same (putative) phenotype for any attribute were identified based on either the empirical *p*-value or *z*-score and data from these line was combined across the 'mol %' and 'nmol/g FW' datasets. Loci from this list were analyzed and loci where > 50% of the sampled lines showed a phenotype at a given cut off are considered putative mutants. To identify lines out of the putative mutants where phenotypic information is known, loci were cross-referenced to information from Aracyc and Gene Ontology annotation on biological processes (for experimentally-derived evidence codes only). Phenotypes predicted for these loci was then compared to phenotypes or experimental evidence reported in the literature to see if the predicted phenotype had been reported or if there was evidence for the gene product to act in a pathway leading directly to or from the measured metabolites.

## Authors' contributions

SMB conceived of and carried out the study, developed the statistical code, performed the statistical analysis and drafted the manuscript. LDB collaborated on methods development, provided statistics code, and performed statistical code quality assurance. RLL participated in design and acquisition of the Chloroplast 2010 Project data and drafted the manuscript. All authors read and approved the final manuscript.

## Supplementary Material

Additional file 1 **This file contains two folders (Code and Data) along with a README file with a brief description of the contents in each folder as well as instructions for execution of code**. The 'Data' folder contains six files representing all the data used in the biological analyses presented, including the Aracyc and Gene Ontology files. The 'Code' folder contains three files representing all the code used to carry out the analyses as well as a Sweave file (MIPHENO.pdf) which illustrates how to carry out the analysis on the Chloroplast 2010 dataset presented in the manuscript. Click here for file
