# Supplementary material for: MIPHENO: data normalization for high throughput metabolite analysis
Source: BMC Bioinformatics. 2012 Jan 13;13:10. doi: 10.1186/1471-2105-13-10 (PMC3278354; doi:10.1186/1471-2105-13-10)
Supplement: Additional file 1 — This file contains two folders (Code and Data) along with a README file with a brief description of the contents in each folder as well as instructions for execution of code. The 'Data' folder contains six files representing all the data used in the biological analyses presented, including the Aracyc and Gene Ontology files. The 'Code' folder contains three files representing all the code used to carry out the analyses as well as a Sweave file (MIPHENO.pdf) which illustrates how to carry out the analysis on the Chloroplast 2010 dataset presented in the manuscript. [file 1471-2105-13-10-S1.ZIP › Additional Files/Code/MIPHENO.pdf]

R MIPHENO Analysis

Shannon M. Bell

1 Introduction

This report details how the analyses presented in the accompanying paper were conducted. The purpose of the document is to illustrate how the functions were used to generate the present results as well as to provide information for the user to modify the process to fit their needs. Functions are described in detail in the included functions files.

2 MIPHENO-Seed data

This section will perform the MIPHENO analysis on seed data collected in the Chloroplast 2010 project. Metabolite data is reported in both mol% and in terms of nmol/gFW and so analysis will be demonstrated on each. First, the required libraries are needed. If you do not know how to install a library, please refer to the R documentation. Set the working directory to the location of the MIPHENO Supplementary Files folder using the setwd("path") command. Then we can bring in all the functions from the MIPHENO_Functions.R file

```
> library(doBy)
> library(gdata)

gdata: read.xls support for 'XLS' (Excel 97-2004) files ENABLED.

gdata: read.xls support for 'XLSX' (Excel 2007+) files ENABLED.

> source("MIPHENO_Functions.R")
> ls()

[1] "both.tail"   "cdf.pval"    "find_hits"   "mad.scores"  "rm.mid"
[6] "rm.outliers"
```

As you can see, there are 6 objects in the working environment, corresponding to the functions in the MIPHENO_Functions.R file The next steps bring in the data and preprocess it. Note that comment (#) lines are process descriptions and not run

```
> data<-read.table("../Data/seedabsolute100811.txt",sep="\t", header=TRUE)
> #Removing known bad C/N data, that persists in the database
> data[,11][data[,10]>80] <-NA
```

```
> data[,10][data[,10]>80] <-NA
> #removing known bad amino acid plates that persist in the database
> badplate<-c('AAS1004', 'AAS1005','AAS1006')
> data[,18][data[,38] %in% badplate]<-NA
> #this fixes the issue with lacking plate dates not having NA
> data[,38][data[,38] == '']<-NA
> #these are lines that are Col-0 but are not annotated as such in the database
> lines<-c("SALK_126784","npq1-2","SALK_039694","SAIL_1250_C12","SAIL_868_G02")
> ex.lines<-subset(data, SOURCEREF %in% lines)
> col0<-subset(data, ECOTYPE == 'Col-0')
> #note that SSB4 is single seed decent from Col-0
> #but has different metabolite profile thus is excluded
> col0<-subset(col0, SOURCEREF != 'SSB4')
> col0<-rbind(col0, ex.lines)
> col0<-drop.levels(subset(col0, select=-ECOTYPE))
> PID<-as.factor(col0$PLATE_ID)
> PD<-as.factor(col0$P_DATE)
> CND<-as.factor(col0$CN_DATE)
> HD<-as.factor(col0$H_DATE)
> qcol0<-cbind(col0[,c(1,2,5:8)],"P_DATE"=PD, "H_DATE"=HD, "CN_DATE"=CND,
+ "PLATE_ID"=PID, col0[,c(9,10,13:35)])
> qcol0<-qcol0[do.call(order, subset(qcol0, select=c(FLATCODE, POTCODE))),]
> rm(PD, PID,CND, HD,badplate, ex.lines, lines)
> colnames(qcol0)

 [1] "LOCUS"      "SOURCEREF"  "POTCODE"    "LABSTOCK"   "GENOTYPING"
 [6] "FLATCODE"   "P_DATE"     "H_DATE"     "CN_DATE"    "PLATE_ID"
[11] "CARBON"     "NITROGEN"   "ALA"        "ARG"        "ASN"
[16] "ASP"        "CYS"        "GABA"       "GLN"        "GLU"
[21] "GLY"        "HPRO"       "HSER"       "HIS"        "ILE"
[26] "LEU"        "LYS"        "MET"        "PHE"        "PRO"
[31] "SER"        "THR"        "TRP"        "TYR"        "VAL"

> #rename prepped data for use later
> seedFW.qcol0<-qcol0
```

The object qcol0 contains the quantitative data for the lines with ecotype Col-0. Attributes of P_DATE (planting date), H_DATE (Harvest date), CN_DATE (Date Carbon/Nitrogen analysis plate was prepared), and PLATE_ID (ID of plate of seeds processed for amino acid analysis) are grouping factors that describe a set of observations that have shared properties. FLATCODE is another shared property, describing a group of individuals that occupied the same physical planting unit. Note the names of the columns, as these are reflected in the column calls further into the process. As in a later section I will need to use this same dataset for calculating zscores, the last line creates a new, better-named object that we will leave in the environment for later. The next step is a quality control step to remove any groups

(based on assay grouping) that should be omitted from further analysis. Ideally these would be removed in-line but for posthoc analysis of data it is often necessary to remove things that look out of the ordinary as we do not have all the information to explain potential causes for the variation. For example, this may reflect groups where the integration for a peak was off in that set or if conditions of growth were changed and a particular measured attribute was differentially altered. Based on the dataset at hand, most of the variation occurs at the level of assay group, so that is the CN_DATE for the Carbon and Nitrogen measures and PLATE_ID for the amino acid measures. Note that this involved the removal of data for an attribute based on the distribution of the sub grouping compared to the rest of the data for the attribute. See the accompanying code for 'rm.outliers' for a detailed description of the function.

```
> #first, order for QC by CN_DATE
> o.cn<-qcol0[do.call(order, subset(qcol0, select=c(CN_DATE, FLATCODE))),]
> rm.cn<-rm.outliers(o.cn[,c(9,11:12)], parameter='CN_DATE', n=3)
> rm.cn<-cbind(o.cn[,1:10], rm.cn[,2:3], o.cn[,13:35])
> #now order for QC by amino acids
> o.aa<-rm.cn[do.call(order, subset(rm.cn, select=c(PLATE_ID, FLATCODE))),]
> rm.aa<-rm.outliers(o.aa[,c(10,13:35)], parameter='PLATE_ID', n=3)
> #this is the output of data set where assay group showing extreem
> #attribute results have been removed
> assay.rm<-cbind(o.aa[,1:12], rm.aa[,2:24])
> rm(rm.cn, o.aa, o.cn, rm.aa, rh.string)
```

As you can see, we are left with an object 'assay.rm', which contains our data with 'poor quality' assay data removed. In looking at how the data set compares, we can see that there are more missing values in assay.rm than in qcol0- these correspond to assay groups where the data was outside the QC threshold

```
> summary(qcol0[,11:14])

     CARBON          NITROGEN            ALA               ARG
 Min.   : 43.18   Min.   :   2.116   Min.   :  71.35   Min.   :  13.99
 1st Qu.: 55.09   1st Qu.:   3.722   1st Qu.: 268.82   1st Qu.:  71.41
 Median : 55.71   Median :   3.912   Median : 380.34   Median :  97.22
 Mean   : 55.81   Mean   :   3.851   Mean   : 489.81   Mean   : 114.79
 3rd Qu.: 56.47   3rd Qu.:   4.054   3rd Qu.: 580.62   3rd Qu.: 132.07
 Max.   : 69.17   Max.   :   5.410   Max.   :4820.52   Max.   :3512.00
 NA's   :2626.00  NA's   :2626.000   NA's   :2363.00   NA's   :2363.00
```

```
> summary(assay.rm[,11:14])

     CARBON          NITROGEN            ALA               ARG
 Min.   : 43.18   Min.   :   2.116   Min.   :  71.35   Min.   :  13.99
 1st Qu.: 55.09   1st Qu.:   3.808   1st Qu.: 262.24   1st Qu.:  70.31
 Median : 55.71   Median :   3.947   Median : 363.55   Median :  94.63
```

```
 Mean   :  55.81   Mean   :   3.933   Mean   : 433.17   Mean   : 106.10
 3rd Qu.:  56.47   3rd Qu.:   4.071   3rd Qu.: 532.94   3rd Qu.: 126.11
 Max.   :  69.17   Max.   :   5.410   Max.   :4820.52   Max.   :1652.87
 NA's   :2626.00   NA's   :3828.000   NA's   :3006.00   NA's   :2818.00
```

```
> par(mfrow=c(3,1))
> plot(qcol0$ALA~qcol0$FLATCODE, ylab='ALA', xlab='FLAT',
+ new.plot=FALSE)
> plot(assay.rm$ALA~assay.rm$FLATCODE, ylab='', xlab='', col=2, add=TRUE,
+ new.plot=FALSE)
> plot(qcol0$ALA~qcol0$PLATE_ID, ylab='ALA', xlab='Assay Group',
+ new.plot=FALSE)
> plot(assay.rm$ALA~assay.rm$PLATE_ID, ylab='', xlab='', col=2, add=TRUE,
+ new.plot=FALSE)
> plot(qcol0$ALA~qcol0$P_DATE, ylab='ALA', xlab='Planting Date',
+ new.plot=FALSE)
> plot(assay.rm$ALA~assay.rm$P_DATE, ylab='', xlab='', col=2, add=TRUE,
+ new.plot=FALSE)
```

The red boxes in Figure 1 are those that remain after the quality control step. Notice on the top plot that there are several sets of 3 flats that are a bit higher (above F10448). In the Chloroplast 2010 data set, 3 flats form an assay group so in cases where the values from all the flats are outside the QC cutoffs, they would all be removed. The bottom panel shows the planting group. Six flats are planted at the same time and are in theory subject to the same environmental conditions (soil, watering, and growth chamber conditions). However, as you can see there are a few boxes where it is only half red. In these cases, one assay group was outside the QC cutoff while the other was ok. It is based on these observations that the QC and the normalization was chosen to be performed at the level of Assay group. The main reason for not performing either of these at the level of flat group was because of small sample size. The maximum number of individuals in a flat is 32, but often due to lack of germination or a high percentage of a different ecotype can bring the number of individuals below 20 and increasing the likelihood that outliers (i.e. putative mutants) could skew the QC and normalization steps.

The next step is to normalize the data. This serves to bring the data in the same space so that comparisons can be made across the dataset. While typically we would include an internal control within each group, or have replication (i.e. n>=3) to gauge the variance, these aspects are often missing from high throughput first pass screening studies. Detailed comments are given for the carbon and nitrogen (CN) data and the same process is followed for amino acid data.

```
> #CN first, using CN_DATE as this is the sample group
> #representing samples processed together
> o.armCN<-assay.rm[do.call(order, subset(assay.rm, select=c(CN_DATE, FLATCODE))),]
> #this generates the Median of each assay group for each attribute measured
> cn.med<-cbind(summaryBy(.~CN_DATE, data=o.armCN[,c(9,11:12)], FUN=median,
+ na.rm=T, keep.names=T))
```

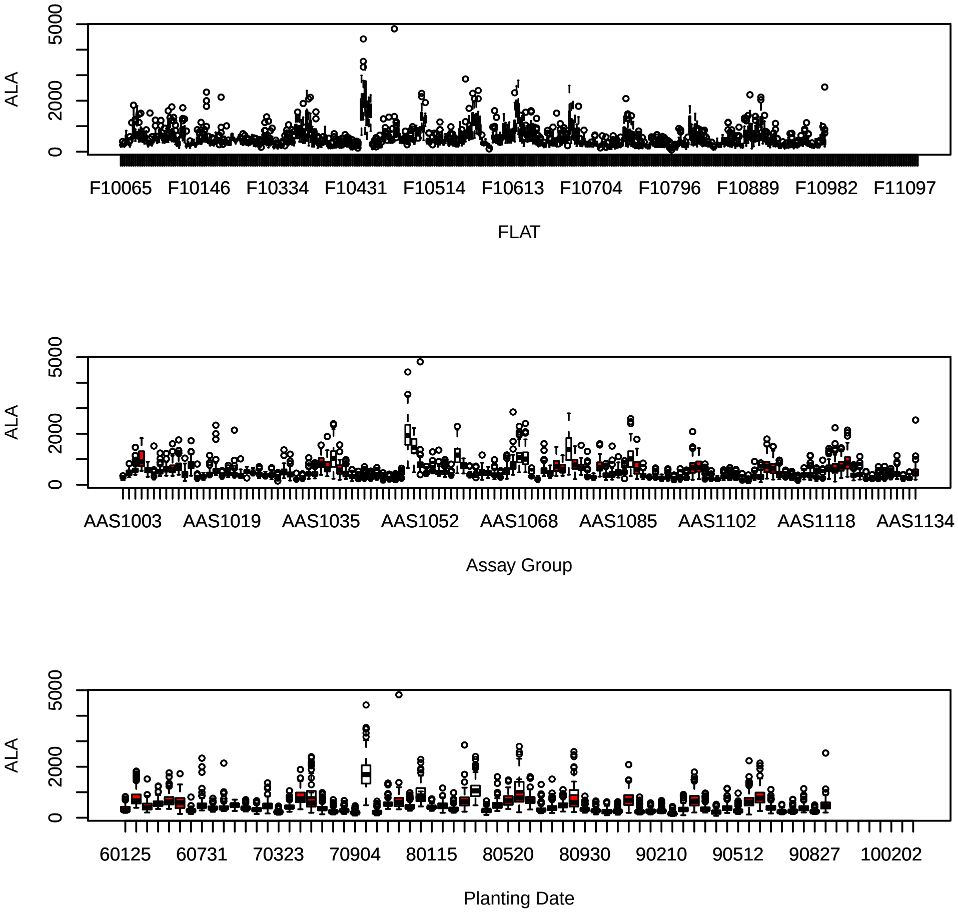

Figure 1: Effect of QC and distribution of data over subunits

```
> #this calculates the overall median for each attribute measured (global median)
> g.cnmed<-apply(o.armCN[,11:12],2,median, na.rm=T)
> #the third column is all that is needed to give the number of samples/group
> #do need to include all so that you dont get off on values when bad sections
> #are removed in the quality control step
> cn.length<-cbind(summaryBy(.~CN_DATE, data=o.armCN[,c(9,11:12)], FUN=length,
+ keep.names=T))
> #this generates a scaling factor for each assay group based on the relationship
> #of assay group median to global median
> cnbet<-sweep(1/cn.med[,2:3],2,FUN='*', g.cnmed)
> ex.cnbet<-cnbet[rep(1:nrow(cnbet),cn.length[,3]),]
> #each observation is then scaled by the scaling factor,
> #found in the object 'ex.cnbet'
> cn.scale<-o.armCN[,11:12]*ex.cnbet
> #the scaled data is then added back into the data frame.
> #ordering the data
> #having a unique id for each observation (POTCODE) help the data remain in order
> cn.scale<-cbind(o.armCN[,1:10], as.data.frame(cn.scale), o.armCN[,13:35])
> #reorder for AA. Applying to PLATE_ID as a plate is extracted and analyzed together
> o.cns<-cn.scale[do.call(order, subset(cn.scale, select=c(PLATE_ID, FLATCODE))),]
> aa.med<-cbind(summaryBy(.~PLATE_ID, data=o.cns[,c(10,13:35)], FUN=median, na.rm=T,
+ keep.names=T))
> g.aamed<-apply(o.cns[,13:35],2,median, na.rm=T)
> aa.length<-cbind(summaryBy(.~PLATE_ID, data=o.cns[,c(10,13:35)], FUN=length,
+ keep.names=T))
> aabet<-sweep(1/aa.med[,2:24],2,FUN='*', g.aamed)
> ex.aabet<-aabet[rep(1:nrow(aabet),aa.length[,3]),]
> aa.scale<-o.cns[,13:35]*ex.aabet
> assay.scale<-cbind(o.cns[,1:12], as.data.frame(aa.scale))
> rm(g.cnmed, cn.med, cn.length, cnbet, ex.cnbet, cn.scale, o.cns, aa.med, g.aamed,
+ aa.length, aabet, ex.aabet, aa.scale, o.armCN, rh.string)
> gc()
```

```
          used (Mb) gc trigger (Mb) max used (Mb)
Ncells 1064840 56.9    1710298 91.4  1710298 91.4
Vcells 5046976 38.6    9973353 76.1  9421621 71.9
```

We are left with an object 'assay.scaled' which contains the data that has been normalized based on the assay group. Figure 2 shows the changes in the data distribution about the median line. As noted in the summary statistics, the median values of the data itself dont change, but the overall spread in each assay group shifts. This is clearly seen based on the placement of the boxes representing the assay groups around the median line (red line, at 363 nmol/gFW ALA).

```
> par(mfrow=c(2,1))
> plot(assay.rm$ALA~assay.rm$PLATE_ID, new.plot=FALSE)
```

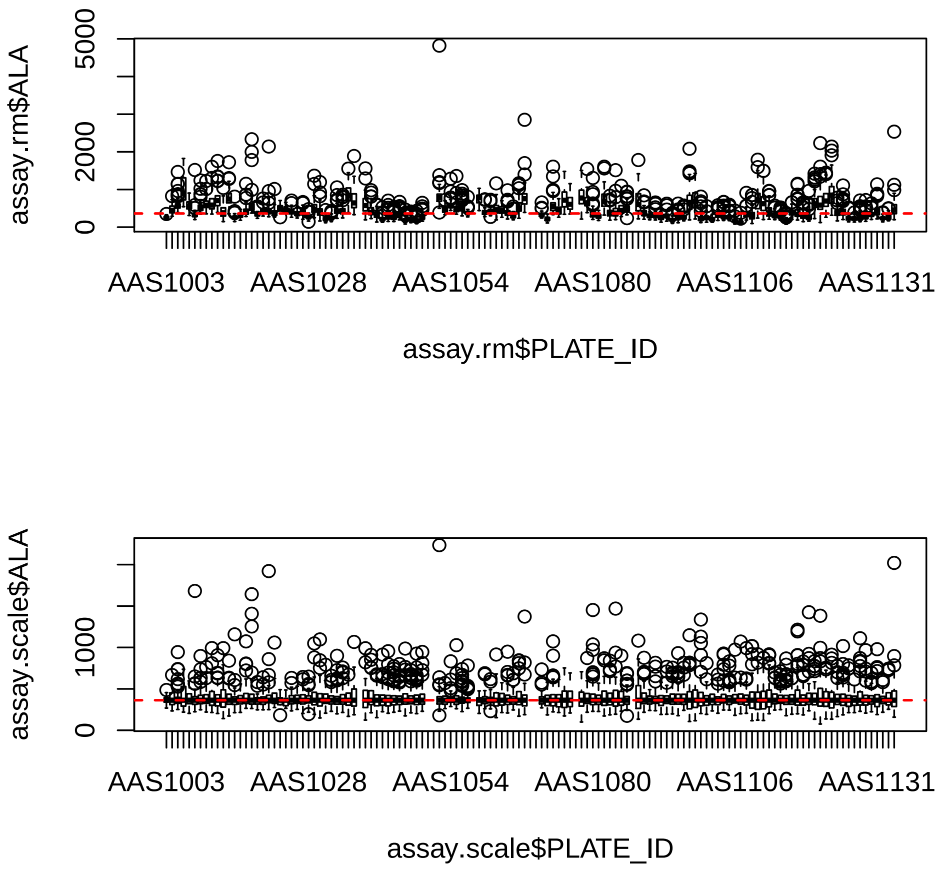

Figure 2: Effect of normalization on data distributions

```
> abline(363,0,col=2, lwd=1.5, lty=2)
> plot(assay.scale$ALA~assay.scale$PLATE_ID, new.plot=FALSE)
> abline(363,0,col=2, lwd=1.5, lty=2)
```

The output of the normalization can be used directly for comparisons across the data set, but as we do not have a true standard to measure what constitutes a 'mutant' phenotype, the ability to compare to other data sets (e.g. for meta analysis) is more limited. An empirical cumulative distribution function (CDF) was chosen as a method of identifying what observations are likely to be considered mutant as it provides a quantitative output that can be adjusted to be more or less stringent. In addition, the CDF can easily be used to identify likely mutants that are higher or low abundance in one step, but taking those approaching zero as likely low abundant mutants and those approaching 1 as likely high abundant mutants. The actual output describes the probability of seeing an observation that extreme or more extreme given the data. See help(ecdf) for more information.

```
> mipheno<-cdf.pval(assay.scale[,11:35])
> mipheno.full<-cbind(assay.scale[,1:10], as.data.frame(mipheno))
```

The object 'mipheno.full' contains the data with empirical p-values. This output file can be used as is for identifying putative mutants, with those having a score ->0 likely to be 'low abundance' mutants and those having a score ->1 likely to be a 'high abundance' mutant. If there was more information about wild type samples, that could have been used to build the NULL distribution (see cdf.pval in MIPHENO_Functions for more information). As all the data was used, you can see that the 1st and 3rd quantile are at 0.25 and 0.75, respectively, which is expected.

```
> summary(mipheno.full[,11:12])
```

```
     CARBON              NITROGEN       
 Min.   :1.011e-04   Min.   :1.151e-04  
 1st Qu.:2.501e-01   1st Qu.:2.501e-01  
 Median :5.071e-01   Median :5.050e-01  
 Mean   :5.002e-01   Mean   :5.001e-01  
 3rd Qu.:7.500e-01   3rd Qu.:7.501e-01  
 Max.   :1.000e+00   Max.   :1.000e+00  
 NA's   :2.626e+03   NA's   :3.828e+03  
```

```
> summary(mipheno.full[,13:14])
```

```
      ALA                 ARG           
 Min.   :1.051e-04   Min.   :1.031e-04  
 1st Qu.:2.501e-01   1st Qu.:2.501e-01  
 Median :5.028e-01   Median :5.023e-01  
 Mean   :5.001e-01   Mean   :5.001e-01  
 3rd Qu.:7.500e-01   3rd Qu.:7.500e-01  
 Max.   :1.000e+00   Max.   :1.000e+00  
 NA's   :3.006e+03   NA's   :2.818e+03  
```

While it is nice to have the whole data file, there were some lines that were run through the pipeline multiple times and some where the information might be widely variable due to the absence of the insertion. The lines screened through the Chloroplast 2010 project are mostly T-DNA insertion lines, which in theory contain an insert within a locus that disrupts gene function. Some of the lines are not homozygous for the insertion and so genotyping was introduced into the pipeline to identify lines that lack the insertion. Therefore, it is useful to remove these lines lacking the insertion before proceeding. Another useful output file (for the analysis presented in the accompanying paper) is the file containing a summary of all the observations for a given insertion. These insertion lines/alleles are identified by the 'SOURCEREF' column in the data frame.

```
> #removing lines with 'Wildtype' or 'Heterozygous' genotyping
> mipheno.ok<-subset(mipheno.full, GENOTYPING %in% c('', 'Homozygous'))
```

```
> #summary emperical p-value by both locus&sourceline
> seedFW<-summaryBy(.~LOCUS + SOURCEREF, data=mipheno.ok[,c(1,2,11:35)], FUN=median,
+ na.rm=T,keep.names=T)
> #cleanup before the next round
> rm(assay.rm, assay.scale, data, col0, mipheno, mipheno.ok, mipheno.full,
+ rh.string, qcol0)
```

The output file 'seedFW' is the MIPHENO empirical p-values for the seed nmol/gFW metabolite data with summary taken over the Locus and the corresponding SOURCEREF (insertion line). The next step is to repeat the process for the other seed data file and for the two leaf files. As the process is the same, the code is given with minimal commenting to aid in generating the output files needed for the next step.

```
> data<-read.table("../Data/seedmolper100811.txt",sep="\t", header=TRUE)
> data[,11][data[,10]>80] <-NA
> data[,10][data[,10]>80] <-NA
> badplate<-c('AAS1004', 'AAS1005','AAS1006')
> data[,18][data[,38] %in% badplate]<-NA
> data[,38][data[,38] == '']<-NA
> lines<-c("SALK_126784","npq1-2","SALK_039694","SAIL_1250_C12","SAIL_868_G02")
> ex.lines<-subset(data, SOURCEREF %in% lines)
> col0<-subset(data, ECOTYPE == 'Col-0')
> col0<-subset(col0, SOURCEREF != 'SSB4')
> col0<-rbind(col0, ex.lines)
> col0<-drop.levels(subset(col0, select=-ECOTYPE))
> PID<-as.factor(col0$PLATE_ID)
> PD<-as.factor(col0$P_DATE)
> CND<-as.factor(col0$CN_DATE)
> HD<-as.factor(col0$H_DATE)
> qcol0<-cbind(col0[,c(1,2,5:8)],"P_DATE"=PD, "H_DATE"=HD, "CN_DATE"=CND,
+ "PLATE_ID"=PID, col0[,c(9,10,13:35)])
> qcol0<-qcol0[do.call(order, subset(qcol0, select=c(FLATCODE, POTCODE))),]
> rm(PD, PID,CND, HD,badplate, ex.lines, lines)
> #rename prepped data for use later
> seedmolper.qcol0<-qcol0
> #quality control
> o.cn<-qcol0[do.call(order, subset(qcol0, select=c(CN_DATE, FLATCODE))),]
> rm.cn<-rm.outliers(o.cn[,c(9,11:12)], parameter='CN_DATE', n=3)
> rm.cn<-cbind(o.cn[,1:10], rm.cn[,2:3], o.cn[,13:35])
> o.aa<-rm.cn[do.call(order, subset(rm.cn, select=c(PLATE_ID, FLATCODE))),]
> rm.aa<-rm.outliers(o.aa[,c(10,13:35)], parameter='PLATE_ID', n=3)
> assay.rm<-cbind(o.aa[,1:12], rm.aa[,2:24])
> rm(rm.cn, o.aa, o.cn, rm.aa, rh.string)
> #normalization
> o.armCN<-assay.rm[do.call(order, subset(assay.rm, select=c(CN_DATE, FLATCODE))),]
```

```
> cn.med<-cbind(summaryBy(.~CN_DATE, data=o.armCN[,c(9,11:12)], FUN=median, na.rm=T,
+ keep.names=T))
> g.cnmed<-apply(o.armCN[,11:12],2,median, na.rm=T)
> cn.length<-cbind(summaryBy(.~CN_DATE, data=o.armCN[,c(9,11:12)], FUN=length,
+ keep.names=T))
> cnbet<-sweep(1/cn.med[,2:3],2,FUN='*', g.cnmed)
> ex.cnbet<-cnbet[rep(1:nrow(cnbet),cn.length[,3]),]
> cn.scale<-o.armCN[,11:12]*ex.cnbet
> cn.scale<-cbind(o.armCN[,1:10], as.data.frame(cn.scale), o.armCN[,13:35])
> o.cns<-cn.scale[do.call(order, subset(cn.scale, select=c(PLATE_ID, FLATCODE))),]
> aa.med<-cbind(summaryBy(.~PLATE_ID, data=o.cns[,c(10,13:35)], FUN=median, na.rm=T,
+ keep.names=T))
> g.aamed<-apply(o.cns[,13:35],2,median, na.rm=T)
> aa.length<-cbind(summaryBy(.~PLATE_ID, data=o.cns[,c(10,13:35)], FUN=length,
+ keep.names=T))
> aabet<-sweep(1/aa.med[,2:24],2,FUN='*', g.aamed)
> ex.aabet<-aabet[rep(1:nrow(aabet),aa.length[,3]),]
> aa.scale<-o.cns[,13:35]*ex.aabet
> assay.scale<-cbind(o.cns[,1:12], as.data.frame(aa.scale))
> rm(g.cnmed, cn.med, cn.length, cnbet, ex.cnbet, cn.scale, o.cns, aa.med, g.aamed,
+ aa.length,aabet, ex.aabet, aa.scale, o.armCN, rh.string)
> gc()

          used (Mb) gc trigger (Mb) max used (Mb)
Ncells  857283 45.8    1710298 91.4  1710298 91.4
Vcells 3923161 30.0   10042946 76.7  9972706 76.1

> #generation of emperical p-values
> mipheno<-cdf.pval(assay.scale[,11:35])
> mipheno.full<-cbind(assay.scale[,1:10], as.data.frame(mipheno))
> #generation of final output file
> mipheno.ok<-subset(mipheno.full, GENOTYPING %in% c('', 'Homozygous'))
> seedmp<-summaryBy(.~LOCUS + SOURCEREF, data=mipheno.ok[,c(1,2,11:35)], FUN=median,
+ na.rm=T,keep.names=T)
> #cleanup before the next round
> rm(assay.rm, assay.scale, data, col0, mipheno, mipheno.ok, mipheno.full,
+ rh.string, qcol0)
```

3 MIPHENO-Leaf data

This section will perform the MIPHENO analysis on leaf data collected in the Chloroplast 2010 project. Metabolite data is reported in both mol% and in terms of nmol/gFW and so analysis will be demonstrated on each. The leaf data files contain measures on the leaf fatty acids and amino acids. After going through the Seed data sets (prior section), all the needed functions should be in the workspace. If not, reload them by going to the first few lines in

the prior section. The data for the leaf analysis is similar to that from the seed and so we can process it much in the same manner.

```
> data<-read.table("../Data/leafabsolute100811.txt",sep="\t", header=TRUE)
> #these are lines that are Col-0 but are not annotated as such in the database
> lines<-c("SALK_126784","npq1-2","SALK_039694","SAIL_1250_C12","SAIL_868_G02")
> ex.lines<-subset(data, SOURCEREF %in% lines)
> #want only Col-0 ecotype
> col0<-subset(data, ECOTYPE == 'Col-0')
> #note that SSB4 is single seed decent from Col-0 but
> #has different metabolite profile thus is excluded
> col0<-subset(col0, SOURCEREF != 'SSB4')
> col0<-rbind(col0, ex.lines)
> col0<-drop.levels(subset(col0, select=-ECOTYPE))
> #making them factor as they are numeric in code-for ease later on
> FAD<-as.factor(col0$FA_DATE)
> PD<-as.factor(col0$P_DATE)
> qcol0<-cbind(col0[,c(1,2,4:8)],"P_DATE"=PD,"FA_DATE"=FAD, col0[,c(23,11:21,24:46)])
> qcol0<-qcol0[do.call(order, subset(qcol0, select=c(FLATCODE, POTCODE))),]
> #this fixes the issue with missing plate dates not having NA
> qcol0[,10][qcol0[,10] == '']<-NA
> #this removed the met plates that are just bad
> plates<-c('AA1082','AA1083','AA1084','AA1085','AA1086')
> qcol0[,37][qcol0[,10] %in% plates]<-NA
> rm(PD, FAD, ex.lines, plates, lines)
> colnames(qcol0)

 [1] "LOCUS"       "SOURCEREF"   "POTCODE"     "LABSTOCK"    "GENOTYPING"
 [6] "PARENT_GENO" "FLATCODE"    "P_DATE"      "FA_DATE"     "PLATE_ID"
[11] "X16_0"       "X16_1d7C"    "X16_1d3T"    "X16_2"       "X16_3"
[16] "X18_0"       "X18_1d9"     "X18_1d11"    "X18_2"       "X18_3"
[21] "X18_2dca"    "ALA"         "ARG"         "ASN"         "ASP"
[26] "CYS"         "GABA"        "GLN"         "GLU"         "GLY"
[31] "HPRO"        "HSER"        "HIS"         "ILE"         "LEU"
[36] "LYS"         "MET"         "PHE"         "PRO"         "SER"
[41] "THR"         "TRP"         "TYR"         "VAL"

> #rename prepped data for use later
> leafFW.qcol0<-qcol0
```

Note the column names as those are used for processing step limits later on. Now that the data has been brought in for the leaf, the quality control step can be carried out. This is done the same was as for the seed data sets accept that we have FA_DATE (fatty acid assay date) instead of CN_DATE. Again, I am saving the prepped data for use later on when I calculate the z-scores

```
> o.fa<-qcol0[do.call(order, subset(qcol0, select=c(FA_DATE, FLATCODE))),]
> #note that default is n=3, may be better to make it 2
> rm.fa<-rm.outliers(o.fa[,c(9,11:21)], parameter='FA_DATE', n=3)
> rm.fa<-cbind(o.fa[,1:10], rm.fa[,2:12], o.fa[,22:44])
> #now order for doing amino acids
> o.aa<-rm.fa[do.call(order, subset(rm.fa, select=c(PLATE_ID, FLATCODE))),]
> rm.aa<-rm.outliers(o.aa[,c(10,22:44)], parameter='PLATE_ID', n=3)
> #this is the output of data set where assays that are extreem have been removed
> assay.rm<-cbind(o.aa[,1:21], rm.aa[,2:24])
> rm(rm.fa, o.aa, o.fa, rm.aa, rh.string)
```

Again, the output object 'assay.rm' contains the metabolite data with the assay groupings not meeting the quality control standards removed. The next step is to carry out the normalization to facilitate cross dataset comparisons. The steps are just as described for the seed dataset and thus only the code is shown.

```
> #FA are first
> o.armFA<-assay.rm[do.call(order, subset(assay.rm, select=c(FA_DATE, FLATCODE))),]
> fa.med<-cbind(summaryBy(.~FA_DATE, data=o.armFA[,c(9,11:21)], FUN=median, na.rm=T,
+ keep.names=T))
> g.famed<-apply(o.armFA[,11:21],2,median, na.rm=T)
> fa.length<-cbind(summaryBy(.~FA_DATE, data=o.armFA[,c(9,11:21)], FUN=length,
+ keep.names=T))
> fabet<-sweep(1/fa.med[,2:12],2,FUN='*', g.famed)
> ex.fabet<-fabet[rep(1:nrow(fabet),fa.length[,3]),]
> fa.scale<-o.armFA[,11:21]*ex.fabet
> fa.scale<-cbind(o.armFA[,1:10], as.data.frame(fa.scale), o.armFA[,22:44])
> #reorder for AA
> o.fas<-fa.scale[do.call(order, subset(fa.scale, select=c(PLATE_ID, FLATCODE))),]
> aa.med<-cbind(summaryBy(.~PLATE_ID, data=o.fas[,c(10,22:44)], FUN=median, na.rm=T,
+ keep.names=T))
> g.aamed<-apply(o.fas[,22:44],2,median, na.rm=T)
> aa.length<-cbind(summaryBy(.~PLATE_ID, data=o.fas[,c(10,22:44)], FUN=length,
+ keep.names=T))
> aabet<-sweep(1/aa.med[,2:24],2,FUN='*', g.aamed)
> ex.aabet<-aabet[rep(1:nrow(aabet),aa.length[,3]),]
> aa.scale<-o.fas[,22:44]*ex.aabet
> assay.scale<-cbind(o.fas[,1:21], as.data.frame(aa.scale))
> rm(g.famed, fa.med, fa.length, fabet, ex.fabet, fa.scale, o.fas, aa.med, g.aamed,
+ aa.length, aabet, ex.aabet, aa.scale, o.armFA, rh.string)
```

The ending object, 'assay.scale' contains the normalized values that are then used to calculate the empirical p-values.

```
> mipheno<-cdf.pval(assay.scale[,11:44])
> mipheno.full<-cbind(assay.scale[,1:10], as.data.frame(mipheno))
```

The nature of the 2010 data is that plants are grown for seed assays first, then some of that seed is planted for leaf assays. This means that genotyping information at the seed stage is potentially available. Thus, we need to look at the genotype value of the parent stock 'PARENT_GENO' as well as the 'GENOTYPING' information of the individual assayed. After the 'ok' lines have been identified, we can take the median phenotype value of the allele over all the assayed measures.

```
> okstock<-unique(subset(assay.scale, PARENT_GENO %in% c('', 'Homozygous'))$LABSTOCK)
> mipheno.ok<-subset(mipheno.full, LABSTOCK %in% okstock)
> #now select only ones with good/potentially ok genotype
> mipheno.ok<-subset(mipheno.ok, GENOTYPING %in% c('', 'Homozygous'))
> gc()

          used (Mb) gc trigger  (Mb) max used  (Mb)
Ncells  873213 46.7    1710298  91.4  1710298  91.4
Vcells 7248012 55.3   13259675 101.2 13258461 101.2

> leafFW<-summaryBy(.~LOCUS + SOURCEREF, data=mipheno.ok[,c(1,2,11:44 )], FUN=median,
+ na.rm=T,keep.names=T)
> #cleanup before the next round
> rm(assay.rm, assay.scale, data, col0, mipheno, mipheno.ok, mipheno.full,
+ rh.string, qcol0)
```

The output object 'leafFW' will be used in subsequent steps. But now we will repeat the process for the mol% data

```
> data<-read.table("../Data/leafmolper100811.txt",sep="\t", header=TRUE)
> lines<-c("SALK_126784","npq1-2","SALK_039694","SAIL_1250_C12","SAIL_868_G02")
> ex.lines<-subset(data, SOURCEREF %in% lines)
> col0<-subset(data, ECOTYPE == 'Col-0')
> col0<-subset(col0, SOURCEREF != 'SSB4')
> col0<-rbind(col0, ex.lines)
> col0<-drop.levels(subset(col0, select=-ECOTYPE))
> FAD<-as.factor(col0$FA_DATE)
> PD<-as.factor(col0$P_DATE)
> qcol0<-cbind(col0[,c(1,2,4:8)],"P_DATE"=PD,"FA_DATE"=FAD, col0[,c(23,11:21,24:46)])
> qcol0<-qcol0[do.call(order, subset(qcol0, select=c(FLATCODE, POTCODE))),]
> qcol0[,10][qcol0[,10] == '']<-NA
> plates<-c('AA1082','AA1083','AA1084','AA1085','AA1086')
> qcol0[,37][qcol0[,10] %in% plates]<-NA
> rm(PD, FAD, ex.lines, plates, lines)
> #rename prepped data for use later
> leafmolper.qcol0<-qcol0
```

And the quality control step

```
> o.fa<-qcol0[do.call(order, subset(qcol0, select=c(FA_DATE, FLATCODE))),]
> rm.fa<-rm.outliers(o.fa[,c(9,11:21)], parameter='FA_DATE', n=3)
> rm.fa<-cbind(o.fa[,1:10], rm.fa[,2:12], o.fa[,22:44])
> o.aa<-rm.fa[do.call(order, subset(rm.fa, select=c(PLATE_ID, FLATCODE))),]
> rm.aa<-rm.outliers(o.aa[,c(10,22:44)], parameter='PLATE_ID', n=3)
> assay.rm<-cbind(o.aa[,1:21], rm.aa[,2:24])
> rm(rm.fa, o.aa, o.fa, rm.aa, rh.string)
```

Now for normalization

```
> o.armFA<-assay.rm[do.call(order, subset(assay.rm, select=c(FA_DATE, FLATCODE))),]
> fa.med<-cbind(summaryBy(.~FA_DATE, data=o.armFA[,c(9,11:21)], FUN=median, na.rm=T,
+ keep.names=T))
> g.famed<-apply(o.armFA[,11:21],2,median, na.rm=T)
> fa.length<-cbind(summaryBy(.~FA_DATE, data=o.armFA[,c(9,11:21)], FUN=length,
+ keep.names=T))
> fabet<-sweep(1/fa.med[,2:12],2,FUN='*', g.famed)
> ex.fabet<-fabet[rep(1:nrow(fabet),fa.length[,3]),]
> fa.scale<-o.armFA[,11:21]*ex.fabet
> fa.scale<-cbind(o.armFA[,1:10], as.data.frame(fa.scale), o.armFA[,22:44])
> o.fas<-fa.scale[do.call(order, subset(fa.scale, select=c(PLATE_ID, FLATCODE))),]
> aa.med<-cbind(summaryBy(.~PLATE_ID, data=o.fas[,c(10,22:44)], FUN=median, na.rm=T,
+ keep.names=T))
> g.aamed<-apply(o.fas[,22:44],2,median, na.rm=T)
> aa.length<-cbind(summaryBy(.~PLATE_ID, data=o.fas[,c(10,22:44)], FUN=length,
+ keep.names=T))
> aabet<-sweep(1/aa.med[,2:24],2,FUN='*', g.aamed)
> ex.aabet<-aabet[rep(1:nrow(aabet),aa.length[,3]),]
> aa.scale<-o.fas[,22:44]*ex.aabet
> assay.scale<-cbind(o.fas[,1:21], as.data.frame(aa.scale))
> rm(g.famed, fa.med, fa.length, fabet, ex.fabet, fa.scale, o.fas, aa.med, g.aamed,
+ aa.length, aabet, ex.aabet, aa.scale, o.armFA, rh.string)
```

Calculate the empirical p-values.

```
> mipheno<-cdf.pval(assay.scale[,11:44])
> mipheno.full<-cbind(assay.scale[,1:10], as.data.frame(mipheno))
```

Get rid of lines with bad genotyping information and generate the output file

```
> okstock<-unique(subset(assay.scale, PARENT_GENO %in% c('', 'Homozygous'))$LABSTOCK)
> mipheno.ok<-subset(mipheno.full, LABSTOCK %in% okstock)
> mipheno.ok<-subset(mipheno.ok, GENOTYPING %in% c('', 'Homozygous'))
> gc()
```

```
          used (Mb) gc trigger  (Mb) max used  (Mb)
Ncells  873364 46.7    1710298  91.4  1710298  91.4
Vcells 8037485 61.4   14002658 106.9 14002623 106.9
```

```
> leafmp<-summaryBy(.~LOCUS + SOURCEREF, data=mipheno.ok[,c(1,2,11:44 )], FUN=median,
+ na.rm=T,keep.names=T)
> #cleanup before the next round
> rm(assay.rm, assay.scale, data, col0, mipheno, mipheno.ok, mipheno.full,
+ rh.string, qcol0)
```

4 Z-score analysis

In order to compare the results of analyzing the data using MIPHENO to the group-based method of z-scores, I needed to calculate the z-scores. As reported in the paper by Lu et al 2008 for the Chloroplast 2010 project, z-scores are calculated on a per-flat basis. The function starts with data that we have already processed in the earlier sections as we can see:

```
> ls()

 [1] "both.tail"        "cdf.pval"         "find_hits"        "leafFW"
 [5] "leafFW.qcol0"     "leafmolper.qcol0" "leafmp"           "mad.scores"
 [9] "okstock"          "rm.mid"           "rm.outliers"      "seedFW"
[13] "seedFW.qcol0"     "seedmolper.qcol0" "seedmp"
```

Note that we have 2 seed data files (seedFW.qcol0, seedmolper.qcol0), and 2 leaf data files (leafFW.qcol0, leafmolper.qcol0) that we will need to calculate the z-scores. There is also a function called 'mad.scores' which will calculate the z-score (named after the median absolute deviation). In keeping with the flow from above, we will start with the seed data and then go onto the leaf. For detailed information on how the function 'mad.scores' works, either type mad.scores() or look into the MIPHENO_Functions.R file. First for the seed nmol/gFW datafile

```
> #calculate the zscore based on the 'FLATCODE'
> zscore.num<-mad.scores(seedFW.qcol0[,c(6,11:ncol(seedFW.qcol0))],
+ parameter='FLATCODE', n=3, out='Zscore')
> #Reattach the attributes, order is be preserved from prior function
> zscore.num.full<-cbind(seedFW.qcol0[,1:10], zscore.num[,2:ncol(zscore.num)])
> summary(seedFW.qcol0[,11:14])

     CARBON           NITROGEN            ALA                ARG
 Min.   :  43.18   Min.   :   2.116   Min.   :  71.35   Min.   :  13.99
 1st Qu.:  55.09   1st Qu.:   3.722   1st Qu.: 268.82   1st Qu.:  71.41
 Median :  55.71   Median :   3.912   Median : 380.34   Median :  97.22
 Mean   :  55.81   Mean   :   3.851   Mean   : 489.81   Mean   : 114.79
 3rd Qu.:  56.47   3rd Qu.:   4.054   3rd Qu.: 580.62   3rd Qu.: 132.07
 Max.   :  69.17   Max.   :   5.410   Max.   :4820.52   Max.   :3512.00
 NA's   :2626.00   NA's   :2626.000   NA's   :2363.00   NA's   :2363.00

> summary(zscore.num.full[,11:14])
```

```
     CARBON               NITROGEN              ALA
 Min.   : -41.07970   Min.   : -14.53987   Min.   :  -3.5065
 1st Qu.:  -0.67449   1st Qu.:  -0.65916   1st Qu.:  -0.6385
 Median :   0.00000   Median :   0.00000   Median :   0.0000
 Mean   :  -0.08953   Mean   :   0.08118   Mean   :   0.2309
 3rd Qu.:   0.67449   3rd Qu.:   0.69036   3rd Qu.:   0.7361
 Max.   :  67.32863   Max.   :  13.91556   Max.   :  36.1532
 NA's   :2626.00000   NA's   :2626.00000   NA's   :2363.0000
      ARG
 Min.   :  -4.3612
 1st Qu.:  -0.6351
 Median :   0.0000
 Mean   :   0.3953
 3rd Qu.:   0.7493
 Max.   : 110.1349
 NA's   :2363.0000
```

You can see that the zscores are in a much different space and that most z-scores are between -1 and 1, as expected.

```
> #NOTE:just want to evaluate pheontypes on ones that are genotyping ok
> z.ok<-subset(zscore.num.full, GENOTYPING %in% c('', 'Homozygous'))
> gc()
```

```
          used (Mb) gc trigger  (Mb) max used  (Mb)
Ncells  873432 46.7    1710298  91.4  1710298  91.4
Vcells 4890332 37.4   14002658 106.9 14002650 106.9
```

```
> seedFWZ<-summaryBy(.~LOCUS + SOURCEREF, data=z.ok[,c(1,2,11:35)], FUN=median,
+ na.rm=T,keep.names=T)
> #cleanup
> rm(zscore.num.full, zscore.num, z.ok)
```

And now for the seed mol% data file

```
> zscore.num<-mad.scores(seedmolper.qcol0[,c(6,11:ncol(seedmolper.qcol0))],
+ parameter='FLATCODE', n=3, out='Zscore')
> zscore.num.full<-cbind(seedmolper.qcol0[,1:10], zscore.num[,2:ncol(zscore.num)])
> z.ok<-subset(zscore.num.full, GENOTYPING %in% c('', 'Homozygous'))
> gc()
```

```
          used (Mb) gc trigger  (Mb) max used  (Mb)
Ncells  873534 46.7    1710298  91.4  1710298  91.4
Vcells 5027798 38.4   14002658 106.9 14002650 106.9
```

```
> seedmpZ<-summaryBy(.~LOCUS + SOURCEREF, data=z.ok[,c(1,2,11:35)], FUN=median,
+ na.rm=T,keep.names=T)
> #cleanup
> rm(zscore.num.full, zscore.num, z.ok)
```

And for the leaf data, same thing accept we have different columns (fatty acid and amino acid data) and there are 2 soures of genotyping information.

```
> zscore.num<-mad.scores(leafFW.qcol0[,c(7,11:ncol(leafFW.qcol0))],
+ parameter='FLATCODE',n=3, out='Zscore')
> zscore.num.full<-cbind(leafFW.qcol0[,1:10], zscore.num[,2:ncol(zscore.num)])
> okstock<-unique(subset(leafFW.qcol0, PARENT_GENO %in% c('', 'Homozygous'))$LABSTOCK)
> z.ok<-subset(zscore.num.full, LABSTOCK %in% okstock)
> z.ok<-subset(z.ok, GENOTYPING %in% c('', 'Homozygous'))
> leafFWZ<-summaryBy(.~LOCUS + SOURCEREF, data=z.ok[,c(1,2,11:44 )], FUN=median,
+ na.rm=T,keep.names=T)
> #cleanup
> rm(zscore.num.full, zscore.num, z.ok, okstock)
> #next the leaf mol% data
> zscore.num<-mad.scores(leafmolper.qcol0[,c(7,11:ncol(leafmolper.qcol0))],
+ parameter='FLATCODE', n=3, out='Zscore')
> zscore.num.full<-cbind(leafmolper.qcol0[,1:10], zscore.num[,2:ncol(zscore.num)])
> okstock<-unique(subset(leafmolper.qcol0, PARENT_GENO %in%
+ c('', 'Homozygous'))$LABSTOCK)
> z.ok<-subset(zscore.num.full, LABSTOCK %in% okstock)
> z.ok<-subset(z.ok, GENOTYPING %in% c('', 'Homozygous'))
> leafmpZ<-summaryBy(.~LOCUS + SOURCEREF, data=z.ok[,c(1,2,11:44 )], FUN=median,
+ na.rm=T,keep.names=T)
> #cleanup
> rm(zscore.num.full, zscore.num, z.ok, okstock)
> ls()

 [1] "both.tail"        "cdf.pval"         "find_hits"        "leafFW"
 [5] "leafFW.qcol0"     "leafFWZ"          "leafmolper.qcol0" "leafmp"
 [9] "leafmpZ"          "mad.scores"       "rh.string"        "rm.mid"
[13] "rm.outliers"      "seedFW"           "seedFW.qcol0"     "seedFWZ"
[17] "seedmolper.qcol0" "seedmp"           "seedmpZ"
```

Now we should have 8 files in the environment containing scores relating to wheter or not observations indicate a putative hit: leafmpZ, leafmp, leafFWZ, leafFW, seedmpZ, seedmp, seedFWZ, seedFW These are going to be used as input for the next step, which is to identify putative hits. First steps are to get all the metabolite data into a single file for a single measure type (e.g. all the mol% data reported as p-values) to reduce the number of data objects. Do note that the Carbon/Nitrogen data will be the same in the mol% as in the FW files.

```
> #reassigning so that same code can be used for all the data types
> leaf<-leafmp
> seed<-seedmp
> #for columns that are the same in both datasets,
> #rename them to identify the data type they are from
```

```
> colnames(leaf)<-c(colnames(leaf[,1:13]),paste('LF_',colnames(leaf)[14:36], sep=""))
> colnames(seed)<-c(colnames(seed[,1:4]),paste('SD_',colnames(seed)[5:27], sep=""))
> #merge the 2 dataframes, NA's will appear of data is missing from one dataset
> mpmerge<-merge(leaf, seed, all=TRUE, by=c('LOCUS', 'SOURCEREF'))
> #now repeat for the nmol/gFW data
> leaf<-leafFW
> seed<-seedFW
> colnames(leaf)<-c(colnames(leaf[,1:13]),paste('LF_',colnames(leaf)[14:36], sep=""))
> colnames(seed)<-c(colnames(seed[,1:4]),paste('SD_',colnames(seed)[5:27], sep=""))
> fwmerge<-merge(leaf, seed, all=TRUE, by=c('LOCUS', 'SOURCEREF'))
> #now for the zscore data
> leaf<-leafmpZ
> seed<-seedmpZ
> colnames(leaf)<-c(colnames(leaf[,1:13]),paste('LF_',colnames(leaf)[14:36], sep=""))
> colnames(seed)<-c(colnames(seed[,1:4]),paste('SD_',colnames(seed)[5:27], sep=""))
> Zmpmerge<-merge(leaf, seed, all=TRUE, by=c('LOCUS', 'SOURCEREF'))
> #now repeat for the zscore nmol/gFW data
> leaf<-leafFWZ
> seed<-seedFWZ
> colnames(leaf)<-c(colnames(leaf[,1:13]),paste('LF_',colnames(leaf)[14:36], sep=""))
> colnames(seed)<-c(colnames(seed[,1:4]),paste('SD_',colnames(seed)[5:27], sep=""))
> Zfwmerge<-merge(leaf, seed, all=TRUE, by=c('LOCUS', 'SOURCEREF'))
> rm(seed, leaf)
> ls()
```

```
 [1] "both.tail"         "cdf.pval"          "find_hits"         "fwmerge"
 [5] "leafFW"            "leafFW.qcol0"      "leafFWZ"           "leafmolper.qcol0"
 [9] "leafmp"            "leafmpZ"           "mad.scores"        "mpmerge"
[13] "rh.string"         "rm.mid"            "rm.outliers"       "seedFW"
[17] "seedFW.qcol0"      "seedFWZ"           "seedmolper.qcol0" "seedmp"
[21] "seedmpZ"           "Zfwmerge"          "Zmpmerge"
```

Now we are left with 4 data objects, two with empirical p-values generated by MIPHENO and two with z-scores. As the same data went into making these, the only differences between the MIPHENO and Z-scores would be if data were thrown out of MIPHENO due to a quality control issue. A 'hit' is described as a 'LOCUS' where at least 2 'SOURCEREF' or alleles for that locus show a phenotype in the same direction. The cutoff (both with empirical p-values and with z-scores) can be adjusted to suite the follow-up capacity. With the function 'find_hits', a parameter must be supplied indicating the type of cutoff that is used (i.e. z-score or empirical p-value). Details can be found in the 'MIPHENO_Functions.R' file. An empirical value of 0.2 was chosen initially for the MIPHENO data, which represents the most extreme 10% in each tail. Based on conversations with people using the Chloroplast 2010 data a z-score of 2.5 was chosen initially as the cutoff, which means any observations <=-2.5 or >=2.5 are considered putative hits. Note that only the quantitative data is analyzed, so that is data contained in columns 3:61. In addition, any line that is not tied to a locus is

removed First, the MIPHENO data is analyzed, then the Z-scores for each data type (mol% or nmol/gFW). The function is processor intensive and tends to take a while.

```
> #remove any samples that are not tied to a locus
> mpmerge<-subset(mpmerge, LOCUS !='')
> fwmerge<-subset(fwmerge, LOCUS !='')
> #I want to go through all loci so source=NULL
> #note that this will take a while depending on how many Loci
> #and the number of attributes
> hit.Mmp<-find_hits(data=mpmerge, source=NULL, values=list(start=3, stop=61),
+ var.cuts=FALSE, cutoff=0.20, Z=NULL)
> gc()

          used (Mb) gc trigger  (Mb) max used  (Mb)
Ncells  876452 46.9    1710298  91.4  1710298  91.4
Vcells 5656576 43.2   14002658 106.9 14002658 106.9

> hit.Mfw<-find_hits(data=fwmerge, source=NULL, values=list(start=3, stop=61),
+ var.cuts=FALSE, cutoff=0.20, Z=NULL)
> gc()

          used (Mb) gc trigger  (Mb) max used  (Mb)
Ncells  876531 46.9    1710298  91.4  1710298  91.4
Vcells 5688290 43.4   14002658 106.9 14002658 106.9

> #and now for the Zscores
> #remove any samples that are not tied to a locus
> Zmpmerge<-subset(Zmpmerge, LOCUS !='')
> Zfwmerge<-subset(Zfwmerge, LOCUS !='')
> hit.Zmp<-find_hits(data=Zmpmerge, source=NULL, values=list(start=3, stop=61),
+ var.cuts=FALSE, Z=2.5,cutoff=NULL)
> gc()

          used (Mb) gc trigger  (Mb) max used  (Mb)
Ncells  876610 46.9    1710298  91.4  1710298  91.4
Vcells 5711982 43.6   14002658 106.9 14002658 106.9

> hit.Zfw<-find_hits(data=Zfwmerge, source=NULL, values=list(start=3, stop=61),
+ var.cuts=FALSE, Z=2.5, cutoff=NULL)
> gc()

          used (Mb) gc trigger  (Mb) max used  (Mb)
Ncells  876687 46.9    1710298  91.4  1710298  91.4
Vcells 5732133 43.8   14002658 106.9 14002658 106.9
```

Lets see what the differences are in the number of putative hits between the outputs

```
> dim(hit.Mmp)
```

```
[1] 470  61
```

```
> dim(hit.Mfw)
```

```
[1] 371  61
```

```
> dim(hit.Zmp)
```

```
[1] 187  61
```

```
> dim(hit.Zfw)
```

```
[1] 180  61
```

Note that there is a clear difference in the number of putative hits between all the datasets, but especially between the MIPHENO scores and the Zscores (indicated by the first value, the number of rows in each data frame). This suggests that the cutoff for Zscores (2.5) is possibly too restrictive. Another thing to note is that more lines come up as putative hits in the mol% data sets. Now I want to combine the data types (nmol/gFW and mol%) to look for the robust hits, i.e. things meeting the criteria for a hit in an attribute across both the data types. There can be some variability in the data and in general, traits that appear as a phenotypic hit in one data set should match in the other. One exception is lines that show many large phenotypes, but they are generally included as at least one attribute has changed. To keep the source of the phenotypic score identifiable, the SOURCEREF (allele) name is appended with 'mp' for values coming from the mol% data and only data from loci represented in the mol% and nmol/gFW sets are investigated.

```
> #to preserve the data that came from the MP dataset, appending MP to the SOURCEREF
> hit.Mmp$SOURCEREF<-paste(hit.Mmp$SOURCEREF,'MP', sep=".")
> #merge to get the data where they both match
> M.locus<-intersect(unique(hit.Mmp$LOCUS), unique(hit.Mfw$LOCUS))
> M.hits<-rbind(subset(hit.Mmp, LOCUS %in% M.locus),
+ subset(hit.Mfw, LOCUS %in% M.locus))
> #order it so it makes an easier to read output
> M.hits<-M.hits[do.call(order, subset(M.hits, select=c(LOCUS, SOURCEREF))),]
> #now i want to make sure that it is a hit given both the mol% and the nmol/gFW
> MIPHENO.hits<-find_hits(data=M.hits, source=NULL, values=list(start=3, stop=61),
+ var.cuts=FALSE, cutoff=0.20, Z=NULL)
> #same for the zscore data
> hit.Zmp$SOURCEREF<-paste(hit.Zmp$SOURCEREF,'MP', sep=".")
> Z.locus<-intersect(unique(hit.Zmp$LOCUS), unique(hit.Zfw$LOCUS))
> Z.hits<-rbind(subset(hit.Zmp, LOCUS %in% Z.locus),
+ subset(hit.Zfw, LOCUS %in% Z.locus))
> Z.hits<-Z.hits[do.call(order, subset(Z.hits, select=c(LOCUS, SOURCEREF))),]
> Zscore.hits<-find_hits(data=Z.hits, source=NULL, values=list(start=3, stop=61),
+ var.cuts=FALSE, Z=2.5, cutoff=NULL)
> dim(MIPHENO.hits)
```

```
[1] 316  61
```

```
> dim(Zscore.hits)
```

```
[1] 196  61
```

```
> #identify the number of unique putative hits, base on # of loci
> length(unique(MIPHENO.hits$LOCUS))
```

```
[1] 68
```

```
> length(unique(Zscore.hits$LOCUS))
```

```
[1] 44
```

In looking at the number of samples that passed through the filter (indicated by the number of unique Loci), there are 68 Loci from MIPHENO and 44 from the Zscores. While some of these might be valid mutants, I am interested in finding out what the false non-discovery rate is between the two analysis techniques. Without a set of lines (ie known mutants in defined Loci) put into the dataset and run in replicates, this can be hard to determine. Instead, I relied on information in the literature to guide the analysis. Data is available in TAIR (The Arabidopsis Information Resource) from their FTP site for both the Gene Ontology and Aracyc (metabolic pathway) information. Some annotations in Aracyc are created from computationally derived information so both datasets were used to try to remove things where the likelihood of finding phenotypic information in the literature is slim. Note that the files were adjusted from TAIR to make them more suitable to the analysis (see the read me file)

```
> go.data<-read.table("../Data/ATH_GO_GOSLIM100811edt.txt", sep='\t', header=TRUE,
+ colClasses="character", fill=TRUE, na.strings='')
> aracyc.data<-read.table("../Data/aracyc_pathways.20100621edt.txt", sep='\t',
+ header=TRUE, colClasses="character", fill=TRUE, na.strings='')
> #here are codes for what I am considering 'experimentally derived' annotation
> evi<-c('IDA', 'EXP', 'IPI','IMP', 'IGI')
> go.data2<-subset(go.data, Aspect != 'C' & Evidence.Code %in% evi)
> #this will get the loci which have the appropriate GO annotation AND
> #are assigned to a pathway
> both.loci<-intersect(go.data2$LOCUS, aracyc.data$LOCUS)
> both.loci<-c(both.loci, 'At4g27030')
> #to get the list of MIPHENO hits for manual checking
> MIPHENO.putative.hits<-subset(MIPHENO.hits, LOCUS %in% both.loci)
> #to get the list of Zscore hits for manual checking
> Z.putative.hits<-subset(Zscore.hits, LOCUS %in% both.loci)
> #lets see what Loci are present in each data set
> unique(MIPHENO.putative.hits$LOCUS)
```

```
 [1] At1g08250 At1g09795 At1g11790 At1g22450 At1g24180 At1g65960 At2g14750
 [8] At2g39800 At3g11170 At3g14420 At3g45300 At3g51240 At4g19710 At4g27030
[15] At4g33150 At5g05730 At5g53460
3520 Levels:  At1g01080 At1g01300 At1g01520 At1g01690 At1g01790 ... At5g64840
```

```
> unique(Z.putative.hits$LOCUS)
```

```
 [1] At1g50480 At1g65960 At2g14750 At2g39800 At2g47240 At3g11170 At3g45300
 [8] At4g19710 At4g27030 At4g33150 At5g05730
3520 Levels:  At1g01080 At1g01300 At1g01520 At1g01690 At1g01790 ... At5g64840
```

The output files, MIPHENO.putative.hits and Z.putative.hits, contain the putative hits to be vetted using the literature. There are 17 putative hits coming out of MIPHENO and 11 from Zscores. Note that FAD4 (At4g27030) was added as it was not in the Aracyc pathway information but is known to have a phenotype in the Chloroplast 2010 dataset using the traditional Chloroplast 2010 cutoffs. For the numbers and values presented in the paper, loci from this list were search on TAIR to find the paper or papers providing the annotation of the biological function or annotation. The information was then used to see if the phenotypes predicted matched what was published. As the Chloroplast 2010 data measured a limited number of metabolites, if something was reasonably close (biosynthetically traceable on a KEGG pathway) to the published observation, it was considered a match. The same criteria were applied to both MIPHENO and Z datasets and the results are in Table 1 of the manuscript. The results were that 12/17 loci identified by MIPHENO had reasonable hits, based on the criteria, while 7/11 loci identifed by Z-scores had resonable hits using the z-score cut off if 2.5. This corresponsed to similar rates in the filtered set (0.70 and 0.64, respectively) but when considering the. As one concern with the difference in number of positives was the Zscore cut off (based on the synthetic data the score seems high), a new Zscore was chosen such that all the 'true positives' identified in the MIPHENO analysis would be identified. Using a new threshold of 1.3, the analysis was repeated.

```
> #Same as before, using a cut off of 1.3
> #first is the individual datasets
> hit.Zmpv1.3<-find_hits(data=Zmpmerge, source=NULL, values=list(start=3, stop=61),
+ var.cuts=FALSE, Z=1.3,cutoff=NULL)
> gc()
```

```
          used (Mb) gc trigger  (Mb) max used  (Mb)
Ncells  987193 52.8    1710298  91.4  1710298  91.4
Vcells 9077354 69.3   14782790 112.8 14782790 112.8
```

```
> hit.Zfwv1.3<-find_hits(data=Zfwmerge, source=NULL, values=list(start=3, stop=61),
+ var.cuts=FALSE, Z=1.3, cutoff=NULL)
> gc()
```

```
          used (Mb) gc trigger  (Mb) max used  (Mb)
Ncells  987272 52.8    1710298  91.4  1710298  91.4
Vcells 9144695 69.8   14782790 112.8 14782790 112.8
```

```
> dim(hit.Zmpv1.3)

[1] 1126   61

> dim(hit.Zfwv1.3)

[1] 960  61
```

Note the number of lines that now pass through the filter. Before it was approx 400 indvidual lines for the MIPHENO but 180 lines for Zscore, with the new zscore cut off it is closer to 1000. The next step is to combine the hits and see which pass the next filter.

```
> hit.Zmpv1.3$SOURCEREF<-paste(hit.Zmpv1.3$SOURCEREF,'MP', sep=".")
> Z.locus<-intersect(unique(hit.Zmpv1.3$LOCUS), unique(hit.Zfwv1.3$LOCUS))
> Z.hitsv1.3<-rbind(subset(hit.Zmpv1.3, LOCUS %in% Z.locus),
+ subset(hit.Zfwv1.3, LOCUS %in% Z.locus))
> Z.hitsv1.3<-Z.hitsv1.3[do.call(order, subset(Z.hitsv1.3,
+ select=c(LOCUS, SOURCEREF))),]
> Zscore.hitsv1.3<-find_hits(data=Z.hitsv1.3, source=NULL,
+ values=list(start=3, stop=61),
+ var.cuts=FALSE, Z=1.3, cutoff=NULL)
> #identify the number of unique putative hits, base on # of loci
> length(unique(MIPHENO.hits$LOCUS))

[1] 68

> length(unique(Zscore.hits$LOCUS))

[1] 44

> length(unique(Zscore.hitsv1.3$LOCUS))

[1] 272
```

Note how many more Loci come through now with the lower Zscore threshold-about 6x the number before. This could result in a far greater number of putative hits that were missed by MIPHENO. To check agains the literature annotated loci I will use the 'both.loci' vector that was generated earlier containing loci with both GO information and AraCyc pathway annotations.

```
> #to get the list of Zscore hits at the 1.3 cut off for manual checking
> Z.putative.hitsv1.3<-subset(Zscore.hitsv1.3, LOCUS %in% both.loci)
> #lets see what Loci are present in each data set
> unique(MIPHENO.putative.hits$LOCUS)

 [1] At1g08250 At1g09795 At1g11790 At1g22450 At1g24180 At1g65960 At2g14750
 [8] At2g39800 At3g11170 At3g14420 At3g45300 At3g51240 At4g19710 At4g27030
[15] At4g33150 At5g05730 At5g53460
3520 Levels:  At1g01080 At1g01300 At1g01520 At1g01690 At1g01790 ... At5g64840
```

```
> unique(Z.putative.hits$LOCUS)

 [1] At1g50480 At1g65960 At2g14750 At2g39800 At2g47240 At3g11170 At3g45300
 [8] At4g19710 At4g27030 At4g33150 At5g05730
3520 Levels:  At1g01080 At1g01300 At1g01520 At1g01690 At1g01790 ... At5g64840

> unique(Z.putative.hitsv1.3$LOCUS)

 [1] At1g07890 At1g08250 At1g09795 At1g11790 At1g24180 At1g50480 At1g55920
 [8] At1g58290 At1g65960 At1g69370 At1g77590 At2g14750 At2g21590 At2g27450
[15] At2g28190 At2g39800 At2g47240 At3g11170 At3g17240 At3g45300 At3g51240
[22] At4g19710 At4g23900 At4g27030 At4g33150 At5g05730 At5g22630 At5g53460
3520 Levels:  At1g01080 At1g01300 At1g01520 At1g01690 At1g01790 ... At5g64840
```

The result is many more loci passing through the filter with the lower Z-score threshold, as expected. When the putative hits were compared to literature/published information though, no new lines were identified. So that means that only 12/28 are considered reasonable hits, a small 43% compared to 68% with the 2.5 cut off. In going into the number of candidates generated, MIPHENO with a 0.1 cut off generated a list of 68 putative hits where as Z-score method using a 2.5 cut off generated a list of 44. Decreasing the z-score cut off to 1.3 (to enable recovery of hits identified by MIPHENO), the list balloons to 272. Now to recover 12 'true positivesÂŠ one must look at 4 times the number of putative hits (272/68). For first pass screening, the goal is typically to identify the maximum number of putative hits while controlling the number of false positives. Given this criteria, MIPHENO looks to be a superior approach.
